# Impact of using glucose as a sole carbon source to analyze the effect of biochar on the kinetics of biomethane production

**DOI:** 10.1038/s41598-024-59313-y

**Published:** 2024-04-15

**Authors:** Marvin T. Valentin, Andrzej Białowiec

**Affiliations:** 1 Department of Applied Bioeconomy, Wrocław University of Environmental and Life Sciences, 51-630 Wroclaw, Poland; 2 Department of Science and Technology, Engineering and Industrial Research, National Research Council of the Philippines, Taguig, Philippines; 3 Department of Agricultural and Biosystems Engineering, Benguet State University, Km. 5, La Trinidad, 2601 Benguet, Philippines

**Keywords:** Biochar, Glucose, Kinetics, Digestion, Biomethane, Environmental biotechnology, Bioenergy

## Abstract

The adaptation of biochar in anaerobic digestion (AD) positively influences the conversion of substrate to biomethane and promotes system stability. This study investigated the influence of biochar (BC) doses (0 to 8 g/L) on the Biochemical Methane Potential (BMP) of glucose during a 60-day AD in a mesophilic batch-type reactor. The first 6.5 weeks of the experimentation were dedicated to the microorganism’s adaptation to the biochar and degradation of organics from the used inoculum (3 phases of the glucose feeding). The last 2 weeks (4th phase of glucose feeding) represented the assumption, that glucose is the sole carbon source in the system. A machine learning model based on the autoregressive integrated moving average (ARIMA) method was used to model the cumulative BMP. The results showed that the BMP increased with the amount of BC added. The highest BMP was obtained at a dose of 8 g/L, with a maximum cumulative BMP of 390.33 mL CH_4_/g-VS added. Likewise, the system showed stability in the pH (7.17 to 8.17). In contrast, non-amended reactors produced only 135.06 mL CH_4_/g-VS and became acidic at the end of the operation. Reducing the influence of carbon from inoculum, sharpened the positive effect of BC on the kinetics of biomethane production from glucose.

## Introduction

The CH_4_ generation through anaerobic digestion (AD) is typically carried out by the consortia of syntrophic microorganisms involved in the digestion process^1^. The efficient electron transfer between participating microorganisms results in better AD performance^1,2^. During secondary fermentation, diffusive electrons are produced and are carried by electron carriers such as H_2_ and formate^2^ characterized as either interspecies hydrogen transfer (IHT) that happens during the syntrophic metabolism of propionate and/or butyrate^3^; or interspecies formate transfer (IFT)^4^. This mode, which requires a medium to facilitate the transfer, is referred to as indirect interspecies electron transfer (IIET)^5^ and mediated interspecies electron transfer (MIET)^6^. Syntrophic microorganisms are important as they maintain stability in pH and regulate volatile fatty acids (VFAs). Excessive VFA accumulations are accompanied by H_2_ accumulation creating the H_2_ partial pressure beyond the range for syntrophic metabolism^7^.

The stability of an AD can be preserved through the addition of carbon materials like biochar (BC). Wang et al., (2018) observed a reduction in the lag phase by 28.57% with methane proportion at 59.8% on biochar amended-reactors^8^. Wang et al., (2021) confirmed the role of BC derived from sawdust for VFAs syntrophic oxidation coupled with lag phase improvement^7^. Li et., (2020) noted a 30% increase in CH_4_ production as a result of BC addition^9^. Remarkable reduction in lag time at 41%^10^; and enhanced chemical oxygen demand (COD) removal at 51%^11^ are among the advantages of biochar addition. BC addition facilitates direct interspecies electron transfer (DIET) between acetogens and methanogens^1,2,12^.

Biochar, a by-product of biomass pyrolysis (< 900 °C) under oxygen-limited conditions^13^ can promote efficient electron transfer by enhancing DIET resulting in improved methane (CH_4_) production^1^. This efficient electron transfer, which enhances CH_4_ formation, is made possible by the conductive properties of BC^1^ and its redox-active moieties^13^. BC being processed from agricultural residues and even free of charge could find its way to increase the sustainability of biogas technology due to its positive impact. Among other factors affecting AD performance, optimum BC dosage is an important consideration as it may reduce CH_4_ production and even worsen the lag phase when overdosed or underdosed^14,15^. Li et al., (2022) noted a remarkable decrease in the lag phase at BC dosage of 5 g/L and consequently, dosage at 10 g/L and 1.0 g/L showed a decline in methane production rate^14^. Dudek et al., (2019) found that maximum biogas production of Brewer’s Spent Grain (BSG) added with BC at higher concentrations (20–25%) decreased from 85.1 to 61.0 dm^3^/g.d.om (dry organic matter)^15^. The efficacy of BC in improving methane production rate, lag phase, and degradation of dissolved organic and volatile fatty acids is affected by its concentration in the AD system^16^. Biomass type is another factor that influences AD performance. Kaur et al., (2020) reported that BC from wheat straw pyrolyzed at 550 °C had the highest cumulative methane yield of 382 L/(kg VS_added_) accounting for a 24% increase in the CH_4_ production relative to the control compared to that from wood and oil seed rape^17^. Furthermore, wheat straw BC was more efficient (41%) in converting volatile solids (VS)^17^.

Different types of feedstocks for AD used previously in the experiments pose that the results of the influence of BC addition to AD on biomethane production are not unambiguous. Usually used feedstock are mixtures of different organic compounds like proteins, carbohydrates, lipids, cellulose, hemicellulose, and others. The obtained results from such a setup are affected by the inhomogeneity of the feedstock. This research employed pure glucose (Gl) as the sole source of carbon for the production of biomethane. This approach was chosen to achieve homogenous AD conditions and minimize the impact of feedstock chemical composition on the results. When Gl is used as a substrate, it forms chemical acidogenic reactions that result in the production of compounds that may include acetate, butyrate, propionate, lactate, and ethanol^18^. During acidogenic reactions, compounds such as propionic acid [Eq. (1)], butyric acid [Eq. 2)], lactic acid [Eq. (3)], and ethanol [Eq. (4)] are hydrolyzed with water to form acetic acid, hydrogen, and carbon dioxide^18^.

1 $$C{H}_{3}C{H}_{2}COOH+2{H}_{2}O \stackrel{\phantom{a}}{\to } C{H}_{3}COOH+3{H}_{2}+C{O}_{2}$$

2 $$C{H}_{3}C{H}_{2}C{H}_{2}COOH+2{H}_{2}O \stackrel{\phantom{a}}{\to } C{H}_{3}COOH+2{H}_{2}$$

3 $$C{H}_{3}CHOHCOOH+2{H}_{2}O \stackrel{\phantom{a}}{\to } C{H}_{3}COOH+HC{O}_{3}+2{H}_{2}$$

4 $$C{H}_{3}C{H}_{2}OH+{H}_{2}O \stackrel{\phantom{a}}{\to } C{H}_{3}COOH+2{H}_{2}$$

Acetate is a pivotal intermediate product during the anaerobic decomposition of organic matter. Its generation and consumption network are quite complex, which almost covers most steps in the AD process. In this way, most of the pathways will lead to the production of acetate, which will be then converted to biomethane according to the reaction in Eq. (5). Additionally, biomethane will be produced by hydrogenotrophic methanogens in the reaction in Eq. (6).

5 $$C{H}_{3}COOH \stackrel{\phantom{a}}{\to } C{H}_{4}+C{O}_{2}$$

6 $$4{H}_{2}+C{O}_{2} \stackrel{\phantom{a}}{\to } C{H}_{4}+2{H}_{2}O$$

Therefore, this study aimed to investigate the influence of different levels of biochar concentration (0, 2, 4, 6, and 8 g/L) on the biomethane potential of the anaerobic digestion of glucose as the only source of carbon.

## Methods

### Substrate, inoculum, and biochar

The biochar was synthesized from dried wheat straw at a temperature of 900 °C for 60 min residence time^1,19^. The properties of the ground wheat straw were analyzed (Table 1). The inoculum was acquired from an existing commercial agricultural biogas plant (1.0 MW_el_) that treats complex substrate (50% food waste and agricultural residues mostly potatoes and sugar beets). The inoculum was stored for 3 days in a room temperature to eliminate background methane production. After this, it underwent filtration through the use of a 1.0 mm strainer to remove remaining fibers and other solid materials like plastics and stones^20^. The suspended liquid was set aside in a climatic chamber (Pollab, model 140/40, Wilkowice, Poland) at 4 °C and used as inoculum for the succeeding BMP experiments. The portion of the inoculum was subjected to chemical and physical analysis. Glucose was used as a carbon source in the BMP experiment. The proportion of the inoculum and glucose was adjusted at inoculum to substrate ratio (ISR) of 2 based on volatile solids (VS)^21–27^.

**Table 1:** The physical and chemical characteristics of the wheat straw, inoculum, glucose, and biochar used in the study.

Parameters	Wheat straw	Inoculum	Glucose	Biochar
Moisture content (MC), %	6.07	95.98	8.83	3.80
Total solids (TS), %	93.93	4.01	91.17	96.21
Volatile solids (VS), %TS	90.85	60.44	99.98	37.42
Ash content (AC), %TS	8.39	39.56	0.02	36.86
Carbon (C), % TS	–	36.76	40.32	–
Hydrogen (H), % TS	–	5.00	6.63	–
Nitrogen (N), % TS	–	5.28	0.23	–
Sulfur (S), % TS	–	1.85	1.49	–
Oxygen (O), % TS	–	11.55	51.31	–

### Physical and chemical analysis

The materials used in the experiment were subjected to proximate and ultimate analysis as detailed in Table 1. The moisture contents (MC), total solids (TS), volatile solids (VS), and ash content (AS) were determined following standard procedure^28^. The CHNS analysis was applied for inoculum and glucose, to calculate the theoretical (stoichiometric) ultimate biomethane yield (uBMY), using a CHNS analyzer (PerkinElmer, 2400 CHNS/O Series II, Waltham, MA, USA) according to 12902:2007. The biochar pH value in the water-extractable fraction was 8.63 ± 0.13 (shaken with deionized water at 1:10, w/v)^29^.

### Experimental setup

The experimental setup followed the procedure previously conducted at the laboratory by Świechowski et al.^30^. An automatic methane potential test system (BPC Instruments AB, AMPTS® II, Lund, Sweden) in serum bottles (500 mL)^30,31^ was used at a mesophilic condition (37 °C). The 400 mL (reactive volume) of the reactor was filled with the mixture comprising 246.5 mL inoculum, 2.6 g VS glucose, and 150 mL nutrient solution equivalent to an ISR of 2. The 150 mL nutrient solution contained (per liter) 0.2 g MgCl_2_.6H_2_O, 1 g NH_4_Cl, 0.1 g CaCl_2_, 0.2 g Na_2_S.9H_2_O, 2.77 g K_2_HPO_4_, 2.8 g KH_2_PO_4_, 0.1 g yeast extract, 5 mL trace element solution, and 2 mL vitamin solution^29,32^. The composition of trace element solution (per liter) was 1000 mg Na2-EDTA.2H_2_O, 300 mg CoCl_4_, 200 mg MnCl_2_.4H_2_O, 200 mg FeSO_4_.7H_2_O, 200 mg ZnCl_2_, 80 mg AlCl_3_.6H_2_O, 60 mg NaWo_4_.2H_2_O, 40 mg CuCl_2_.2H_2_O, 40 mg NiSO_4_.6H_2_O, 20 mg H_2_SeO_4_, 200 mg HBO_3_ and 200 mg NaMoO_4_.2H_2_0^32^. Vitamin solution consisted of (per liter) 10 mg biotin, 50 mg Pyridoxin HCl, 25 mg Thiamine HCl, 25 mg D-Calcium pantothenate, 10 mg Folic acid, 25 mg Riboflavin, 25 mg Nicotinic acid, 25 mg P-aminobenzoic acid and 0.5 mg vitamin B1^32^. During the AD process, the mixtures were stirred every hour for 3 min using the default mixing setting of the AMPTS. This was to avoid digestion inhomogeneity^31^.

The mixture of glucose and inoculum in the reactor followed an inoculum-to-substrate ratio of 2.0 based on a volatile solid. There were 15 bioreactors used in the experiments which represent triplicates of both the treatment and the control. Reactors 1 to 12 were filled with the same amount of inoculum and substrate and were dosed with biochar at concentrations of 2, 4, 6, and 8 g/L, respectively, while reactors 13, 14, and 15 served as control (blank reactors)—without biochar.

### Substrate loading strategy

The reactors were loaded with glucose four times (Supplementary Fig. S1) throughout the 60-day (day 0, 8, 21, and 44) experimental period to adapt microorganisms to the glucose as a sole source of carbon. Initially, in the reactors, there were two carbon sources namely; the inoculum and glucose. In that case, the inoculum carbon, originating from AD biogas plants receiving 50% food waste and agricultural residues mostly potatoes and sugar beets, could disrupt the real influence of biochar on glucose AD. Therefore, to eliminate the inoculum carbon source special procedure had been designed. First, the theoretical ultimate biomethane potential (uBMP) mL CH_4_/g-VS of the glucose was estimated using the Buswell and Mueller stoichiometric formulas Eq. (7). The yield in volume per unit mass of glucose or inoculum was further calculated using Eq. (8).

7 $${C}_{c}{H}_{h}{O}_{o}{H}_{n}{S}_{s}+\left(c-\frac{h}{4}-\frac{o}{2}+\frac{3n}{4}+\frac{s}{2}\right){H}_{2}O \stackrel{\phantom{a}}{\to } \left(\frac{c}{2}+\frac{h}{8}-\frac{o}{4}-\frac{3n}{8}-\frac{s}{4}\right)C{H}_{4}+\left(\frac{c}{2}-\frac{h}{8}+\frac{o}{4}+\frac{3n}{8}+\frac{s}{4}\right)C{O}_{2}+nN{H}_{3}+s{H}_{2}S$$

8 $${C}_{c}{H}_{h}{O}_{o}+\left(c-\frac{h}{4}-\frac{o}{2}\right){H}_{2}O \stackrel{\phantom{a}}{\to } \left(\frac{c}{2}-\frac{h}{8}+\frac{o}{4}\right)C{O}_{2}+\left(\frac{c}{2}+\frac{h}{8}-\frac{o}{4}\right)C{H}_{4}$$

where, ${C}_{c}{H}_{h}{O}_{o}{H}_{n}{S}_{s}$ are the elemental composition of the biomass that are comprised of carbon (C), hydrogen(H), oxygen(O), nitrogen(N), and sulfur(S); $c, h, o, n, s$ denote the percentage share of the volatile solids of biomass. Hence, the complete degradation of 1.0 g VS of glucose, a theoretical quantity of biogas can reach 746.6 mL/g-VS, with the uBMP at the level of 377.9 mL/g-VS. With this, the 2.6 g VS glucose used in the experiment theoretically had a uBMY of 982.6 mL CH_4_. Likewise, the same was applied with the inoculum as it still exhibits organic matter indicated in the CHNSO analysis where it contains 36.7% of C (Table 1) that could contribute to the overall theoretical biomethane yield. The inoculum had an estimated theoretical uBMP of 594.1 mL CH_4_/g-VS, and considering the 5.2 g of VS the uBMY of inoculum in the reactor should be 3,089.74 mL CH_4_. In total, the uBMY of the mixture should be 4,072.4 mL CH_4_.

Initially, the theoretical biomethane yield from inoculum was 3,089.74 mL CH_4_. Therefore, the first 3 phases were designed to eliminate the influence of the inoculum carbon. Based on the previous experiment^33^, the constant rate (*k*) of biomethane production from the inoculum was 0.13 per day and was used to calculate the accumulation of the biomethane within the time with the application of the first-order equation (Eq. 9).

9 $${BMY}_{t}=uBMY \cdot \left(1-{e}^{\left(-k\cdot t\right)}\right)$$

where BMY_t_ is the cumulative biomethane yield (mL CH_4_) at a given time *t*, uBMY is the ultimate biomethane potential yield (3,089.74 mL CH_4_), *k* is the first-order production rate (0.13 d^-1^), and *t* is the processing time in days. The simulated values were compared with the ultimate biomethane yield. The percentage of biomethane production of the ultimate biomethane yield was calculated.

The prediction indicated that 99% of the uBMY was achieved after 36 days (Supplementary Fig. S2). Additionally, to ensure that all organic matter originating from inoculum was decomposed, an additional 2 weeks were allocated for finalizing the digestion. Therefore, the fourth phase, where only one source of carbon was glucose, and the microorganisms were adapted to the glucose due to the first 3 feeds (the addition of glucose during the first 3 feeds was made when all reactors had reached a biomethane production rate of less than 0.1 mL CH_4_/hr.), started on the 44^th^ day of the experiment, and lasted for 2 weeks.

### Model for data fit

A machine learning model based on the autoregressive integrated moving average (ARIMA) method was used to model the development of BMY. The data was divided into three sets: 70% for training, 20% for validation, and 10% for prediction. Python 3.11 was used as the programming language while Jupyter Notebook (Anaconda 3) as the integrated development environment (IDE) for the code preparation. The model performance was assessed using statistical parameters such as the Akaike Information Criterion (AIC), RMSE, and R^2^ as described in Supplementary Table S1. Additional parametric calculations of the non-linear models were performed to determine the “model efficiency coefficient” of the models^34^.

The modified Gompertz equation was used to interpret the trend of the BMP_t_ development for the 4^th^ phase in the AD of glucose^35–37^ (Eq. (10)). The variables in the model were estimated with the use of Python 3.11 and Statistica 13.0 software (TIBCO Software Inc., Palo Alto, CA, USA).

10 $${BMP}_{t}=bBMP exp\left[-exp\left(\frac{{R}_{max} x e}{P} x \left(\lambda -t\right)+1\right)\right]$$

where, BMP_t_ is the cumulative biomethane potential in mL CH_4_/g-VS at a time $t$; $bBMP$ is the biochemical biomethane potential in mL CH_4_/g-VS at the infinity; ${R}_{max}$ is the maximum biomethane production rate in mL CH_4_/g-VS-d; $\lambda$ is the lag phase in day; and $e$ is a constant (2.71).

## Results and discussions

### Cumulative biochemical methane yield

The cumulative BMY of glucose as influenced by the addition of BC at different concentrations (2, 4, 6, and 8 g/L) over the 60 days AD is shown in Supplementary Fig. S1. The data on BMY_t_ was recorded every 15.0 min and had 5,760 cases for each reactor with a total of 86,400 cases for the 15 reactors. Overall, the addition of biochar improves the BMY compared to the reactors without biochar. Likewise, the cumulative BMY was observed to increase with BC concentration (Table 2). This positive observation of the influence of biochar is highly consistent with previous works reported elsewhere^14,38–41^. In particular, reactors doped with BC at 8 g/L had the highest cumulative BMY followed by the reactors that received BC concentrations of 6, 4, and 2 g/L. The reactors with no biochar addition had the lowest BMY_t_ production throughout the experiment. In terms of stability, as indicated by the monitoring of the pH, supplementation of BC was able to maintain the optimum pH range among the reactors. In particular, at 8 g/L of BC, the pH was stable with an initial value of 7.17 and increased to 8.17 at the end of the process. Lower concentrations showed pronounced fluctuation and a significant drop in the pH at the end of the AD experiment. The non-amended reactors became acidic having a pH of 4.4 at the end of the operation.

**Table 2:** The fitness criterion of the ARIMA model on the BMY from glucose at different BC concentrations.

BC dosage, g/L	Cumulative BMY, mL		(*p, d, q*)	Statistical parameters		
	Actual	Predicted		RSME	R^2^	AIC
0	1036.06	1036.06	7, 1, 7	0.06	0.99	2488.79
2	2360.55	2360.53	7, 1, 5	0.01	0.99	− 7518.54
4	2439.96	2439.94	3, 1, 5	0.01	0.99	− 6749.15
6	2590.20	2590.20	7, 1, 6	0.01	0.99	− 5105.14
8	2676.92	2676.92	4, 1, 6	0.02	0.99	− 5827.92

The BMP from phases 1 to 4 from the ARIMA model and the resulting fits are provided in Supplementary Fig. S3. The assessment of the model in terms of ARIMA order (p, d, q) and statistical indicators is shown in Table 2. Overall, the model for each BC concentration had a high coefficient of determination (> 0.99) and low root mean square error.

### The biomethane production rate fluctuations

#### The biomethane production rate fluctuations during the first phase of the experiment

The impact of biochar addition in terms of biomethane production rate (Supplementary Fig. S4) was mainly on the first peak of the biomethane production rate which could be attributed to easily biodegradable compounds as a result of the glucose degradation. As presented in Supplementary Fig. S4, the glucose degradation rate was highest during day 1. Compared to similar experiments, peaking was reported at a later time such as after day 6 as in the study of Li et al.^14^. The early peak of the biomethane production rate observed in this study could be associated with the characteristics of glucose, being a simple substrate and easily biodegradable. The reactors doped with biochar had a higher biomethane production rate compared to the non-amended reactor. The later peaks could be attributed to other organic compounds present in the inoculum. The peaks in biomethane production rates were observed to be highest at 43.29, 44.83, 27.17, and 39.15 mL/hr at 8, 6, 4, and 2 BC g/L, respectively. In contrast, the lowest biomethane production of 14.25 mL/hr was observed from 0 g/L.

#### The biomethane production rate fluctuations in the second phase of the experiment

During the second phase, the biomethane production rates in all reactors, except for the blank reactors, were highest on day 1 as shown in the Supplementary Fig. S5. The reactors that received 8 g/L of biochar had the highest degradation rate of 36.97 mL/hr which gradually decreased to 20.72 and 8.92 mL/hr at concentrations of 2 and blank reactors, respectively. This indicates that biochar addition at higher concentrations improves the BMP rate. Succeeding biomethane production rate peaks occurred on days 3, 5, 6, and 10 across all reactors, but at lower rates, which could have originated from the degradation of organics present in the inoculum. During the spikes in the biomethane production rates on days 3 and 5, the 8 g/L concentrated reactors had the highest degradation rates with respective values of 7.74, and 5.18 mL/hr while the highest biomethane production rate of 4.51 mL/hr on day 6 was obtained from 4 g/L. The blank reactors exhibited the highest biomethane production rate on days 5 and 10 at 8.8 and 8.9 mL/hr, respectively. However, these values are still significantly lower than the biomethane production rates of the biochar-amended reactors during the first day. This suggests that biochar addition facilitated faster degradation of the glucose as compared to the delayed reaction in the control reactors. Li et., (2021) observed a similar trend, with the amended reactors reaching their peak on day 4, whereas the BMP rate for the control reactors manifested on day 15^42^.

#### The biomethane production rate in the third phase of the experiment

The degradation rate during the third phase is shown in Supplementary Fig. S6. Consistent with the previous phases, a similar trend was observed, with the highest biomethane production rate occurring in all biochar-amended reactors on the first day of the operation. Among the treated reactors, the highest biomethane production rate of 66.72 mL/hr was obtained from 8 g/L. In contrast, the non-biochar reactors exhibited the lowest biomethane production rate of 29.40 mL/hr, significantly lower than that of the biochar-amended reactors. This observation reinforces the earlier findings, highlighting the substantial enhancement of biomethane production rates with biochar addition during the anaerobic digestion of glucose. Furthermore, the peaks in the biomethane production rate on the 5^th^, 9^th^, and 17^th^ days were lower compared to the initial and subsequent phases, indicating the gradual degradation of the organics originating from inoculum.

#### The biomethane production rate in the fourth phase of the experiment

The degradation rate of the organic matter, influenced by the different BC concentrations during the fourth phase, is depicted in Supplementary Fig. S7. The highest biomethane production rate of 22.77 mL/hr was observed at a concentration of 8 g/L, which decreased to 11.85 g/L at 2 g/L, while the blank reactors yielded the lowest biomethane production of 8.62 mL/hr. These peak rates were observed to occur only on day 1. However, the blank reactors showed second peak reaching 2.31 mL/hr on day 2, possibly attributed to delayed reactions from the previous phases and was only reflected in the fourth phase. At this phase, all reactors showed a gradual decrease in the biomethane production rate as compared to the previous 3 phases where multiple peaks were reflected in the graph. This indicates that glucose only served as the sole carbon source during the fourth phase.

### Effects of BC on kinetics of biomethane production from glucose

**Figure 1:**
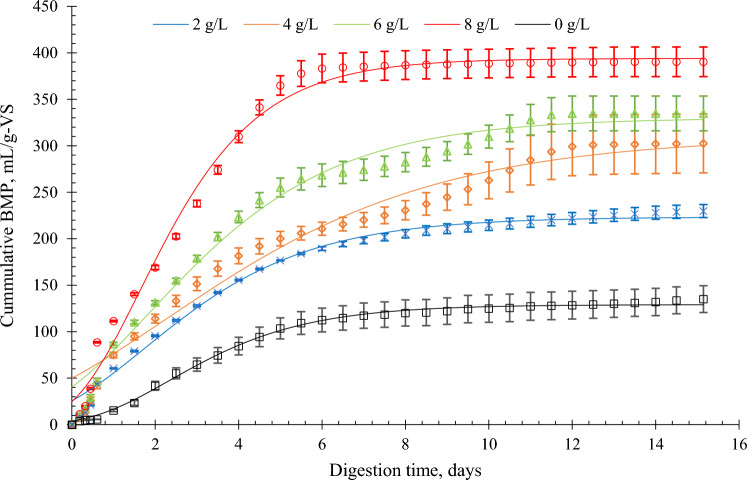
The BMP of glucose influenced by different concentrations of biochar during the fourth phase of the experiment.

The cumulative BMP at different biochar concentrations during the fourth phase of the experiment is presented in Fig. 1. In the final phase of the experiment, where the only source of carbon was glucose, the overall AD performance showed a significant difference in the cumulative BMP across all reactors.

The cumulative and average BMP and the fitness criterion including the kinetic model parameters of the models using the modified Gompertz equation during the fourth phase of the experiment are summarized in Table 3. The highest cumulative BMP, reaching 390.33 mL/ g-VS was achieved at a concentration of 8 g/L. The obtained result aligns closely to the findings of Kaur et al., (2020), who reported a cumulative BMP of 381.92 L/kg VS_added_ at a biochar concentration of 10 g/L^17^. This consistency was also noted by Namal (2020) in a study consisting glucose as a substrate^36^. Ma et al., (2020) reported the same observation that methane production increased with biochar concentration; however, no significant increase was observed with concentrations raging from15 to 20 g/L^43^. The same was attested by Li et al.^14^ that cumulative methane yield subsequently decreased with too much biochar. Zhang et al., (2020) reported a 55.86% cumulative methane yield at 1.5 g biochar addition over the control^44^.

**Table 3:** The BMP performance, fitness criterion, and kinetics parameters from the Modified Gompertz during the fourth phase of the AD of glucose at different BC concentrations (letters in superscripts indicate the statistically significant differences p < 0.05).

BC dosage, g/L	Cumulative BMP, mL/g-VS	Fitness criterion			Kinetic model parameters			
		RSME	R^2^	AIC	_b_BMP, mL/g-VS	${R}_{max}$ mL g-VS/day	k, d^−1^	λ, day
2	229.76^a^	5.14	0.99	4732.12	225.04	38.27	0.17	0.42^b^
4	302.56^a^	11.31	0.98	6321.89	372.05	47.39	0.12	0.47^b^
6	334.71^b^	12.88	0.98	7133.37	381.13	59.95	0.15	0.51^b^
8	390.33^c^	11.27	0.98	7021.09	394.16	90.97	0.23	0.10^a^
0	135.06^a^	4.73	0.91	4384.94	128.82	26.32	0.20	0.76^b^

**Figure 2:**
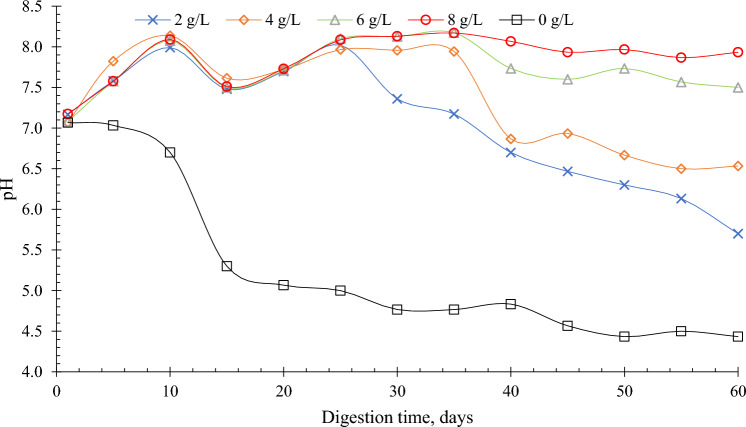
The pH variation due to biochar addition measured from the reactors throughout the experimental period.

The difference in the BMP from 0, 2, and 4 g/L was statistically insignificant. The regression coefficient for reactors with biochar was all above 0.98 indicating a good fit of the experimental data to the modified Gompertz equation. The result of the modified Gompertz estimate shows that the BMP rate was highest at 8 g/L (394.16 mL/day) and with the shortest lag phase of 0.10 days. The monitoring of the pH development during the experiment showed that a dose of BC g/L stabilized the pH at 7.17 to 8.17. Compared to lower concentrations where pH significantly fluctuated and decreased at the end of the operation (Fig. 2). This explains the better BMP production at 8 g/L compared to the other reactors at lower biochar concentrations. The non-amended reactors (0 g/L) had the lowest cumulative BMP of 135.06 mL/g-VS and the longest lag phase of 0.76 days. At 0 g/L the initial pH was 7.1 and it became acidic with a pH of 4.4 at the end of the process. Supplementation of biochar was able to reduce the lag phase in this study which was also observed elsewhere^1,10,17,19^. Jang et al.^38^ reported 2.08 days lag phase at 0 g/L while 1.87 and 1.5 days from biochar addition at 1 and 10 g/L, respectively^38^. Sunyoto et al.^10^ observed 41% lag phase reduction through biochar addition in the AD of carbohydrates food waste^10^.

The highest biomethane production constant rate ($k=0.23/day$) was obtained from reactors having a BC concentration of 8 g/L. Compared to previous phases, the degradation rates were lower in the amended reactors. Likewise, the methane production has decreased in the fourth phase. This decline is attributed to the complete degradation of the organics from the inoculum, as evidenced by the decreased BMP rates from the control reactors. Furthermore, the degradation of glucose starting from the initial phase may have led to acid buildup, reflected in the gradual decrease in pH observed across all reactors, particularly during the fourth phase, potentially contributing to reduced methane production. The excess build-up of volatile fatty acids (VFA) in the reactor usually leads to a pH drop and failure of the system45. In contrast, the control reactors became acidic, reaching a pH of 4.4, while the amended reactors were still in the optimum pH range, though they declined from their original pH levels. This suggests that the biochar that was added in the previous phases facilitated microbial acclimation, resulting in controlled acidification and enhanced buffering capacity of the reactors. Hence, this highlights that microorganisms should be adapted to biochar additions before batch BMP tests of different feedstocks.

To reflect the importance of the experimentation on a single source of carbon, the relative increase of the cumulative BMY from the 60 days, and only from BMP from the 4^th^ phase was determined (Table 4). In the case of the comparison between variants without BC and variants with increasing doses of BC, similar relative increases were found between 56.09 and 61.28%, and between 41.21 to 65.39%, respectively. However, in the comparison between variants containing different BC doses, the higher influence of BC may be observed when data from the 4th phase (with glucose as the only source of carbon) were used. The relative increase of BMP varied between 9.59 to 41.12%, while in the case of the data from cumulative BMY from the 60 days varied from 3.24 to 11.82. It may bring new insight, that for the evaluation of the BC influence on AD performance, better results should be expected in the case of using single organic compounds, as the only source of carbon, instead of using mixtures.

**Table 4:** The relative increase in the cumulative BMY from the 60 days and the BMP from the 4th phase of the experiment.

BC dose rate g/L	0	2	4	6	8
The relative increase of the cumulative BMY from the 60 days					
0	–				
2	56.09	−			
4	57.52	3.25	–		
6	59.98	8.87	5.80	−	
8	61.28	11.82	8.85	3.24	–
The relative increase of the cumulative BMP from the 4th phase					
0	−				
2	41.21	-			
4	55.35	24.06	–		
6	59.64	31.34	9.59	–	
8	65.39	41.12	22.47	14.25	–

### pH Variation

The activity of microbial enzymes and the acid–base balance in the digestion system is reported to be greatly affected by the optimum pH range (6.5 to 7.5) inside the digester Li et al.^46^. In this study, the addition of biochar mitigated the pH variation (Fig. 2). The pH from the non-amended reactor gradually decreased during the first 10 days from 7.1 to 6.7 then sharply decreased to 5.1 at the end of day 20 afterward the pH again gradually decreased until it reached 4.4 at the end of the process. The initial pH from biochar-amended reactors ranged from 7.1 to 7.2 and gradually increased to day 10 at a range of 7.9 to 8.07. At day 15, all reactors' pH slightly dropped and recovered at day 25. The pH at 8 g/L almost remained constant reaching a pH of 7.9 toward the end of the experiment. For the 6 g/L the pH dropped to 7.6 on day 45 then it attempted to recover at day 50 which had a pH of 7.73 and it decreased to 7.5 on day 60. The same with 4 g/L, its pH sharply dropped to 6.8 on day 40 the slightly recovered to 6.9 on day 45 but gradually dropped to 6.5 at the end of the process. Overall, the addition of biochar 8 g/L helped maintain the pH close to neutral. The addition of biochar can maintain alkalinity and stability in AD throughout the process^43,47^.

## Summary

The influence of the different concentrations of biochar at 0, 2, 4, 6, and 8 g/L was investigated in the anaerobic digestion of simple organics represented by glucose in a batch test setup. Overall, the addition of biochar enhanced the BMP and shortened the lag phase compared to the non-amended reactors. In particular, higher biochar concentration had better cumulative BMP and shorter lag time. At a concentration of 8 g/L, the BMP was 390.33 mL^/^g-VS and was significantly higher than other concentrations. Additionally, the constant rate of biomethane production was the highest with 8 g/L of BC and counted at 0.23 d^-1^. The addition of biochar helped stabilize the system by maintaining a pH close to neutral compared to 0 g/L where it became acidic indicating severe inhibition during the process. The experiment revealed, that after 6.5 weeks the glucose-originating carbon is the sole source of carbon for adapted microorganisms, which led to a clearer picture of the influence of biochar on biomethane production than in the case of the system containing both carbon from glucose and inoculum. It shows that the BMP test on the influence of biochar on the AD performance of specific organic compounds requires a longer period of adaptation even if easily biodegradable feedstock is used. Results showed the significant role of biochar in pH stabilization due to the increase of buffering capacity during long-period experiments, while a system without biochar suffers a lack of buffering capacity due to organic matter from inoculum gradual decomposition.

## Supplementary Information

Supplementary Information.

## Data Availability

The datasets used and/or analysed during the current study are available from the corresponding author on reasonable request.
