# Supplementary material for: Impact of using glucose as a sole carbon source to analyze the effect of biochar on the kinetics of biomethane production
Source: Sci Rep. 2024 Apr 15;14:8656. doi: 10.1038/s41598-024-59313-y (PMC11384929; doi:10.1038/s41598-024-59313-y)
Supplement: Supplementary file 1 — Supplementary Information. [file 41598_2024_59313_MOESM1_ESM.docx]

- 1. Supplementary Table

Table S1. Fitness criteria for the BMP of glucose adapted from Pererva et al., (2020)^1^.

| **Criterion** | **Calculation** | **References** |
| --- | --- | --- |
| Akaike Information Criterion | $AIC= \left\{ {N.\ln\left( \frac{RSS}{N} \right)+2M+\frac{2M(M+1)}{N-M-1} \atop N.\ln\left( \frac{RSS}{N} \right)+2M} \right. {\frac{N}{M}<40 \atop\frac{N}{M}\geq40}$ | ^2^ |
| Coefficient of Determination (R^2^) | $R^{2}=1-\frac{\sum_{i=1}^{N} \left( y_{i}-\hat{y}_{i} \right)^{2}}{\sum_{i=1}^{N} \left( y_{i}-\bar{y}_{i} \right)^{2}};$ $=1-\frac{RSS}{\sum_{i=1}^{N} \left( y_{i}-\bar{y}_{i} \right)^{2}}$ | ^1^ |
| Residual Sum of Squares (RSS) | $RSS=\sum_{i=1}^{N} \left( y_{i}-\hat{y}_{i} \right)^{2}$ | ^3^ |
| Root Mean Square Error (RMSE) | $RMSE= \sqrt{\frac{\sum_{i=1}^{N} \left( y_{i}-\hat{y}_{i} \right)^{2}}{N}}$ | ^4^ |

- 1. Supplementary Figures

Fig. S1. The cumulative biomethane yield (NmL) of glucose influenced by the concentrations of biochar.

99% of uBMY after 36 days

Fig. S2. The prediction of the BMY_t_ and percentage of uBMY achieved from inoculum during the time. The indication of 99% of the uBMY achieved after 36 days has been given.


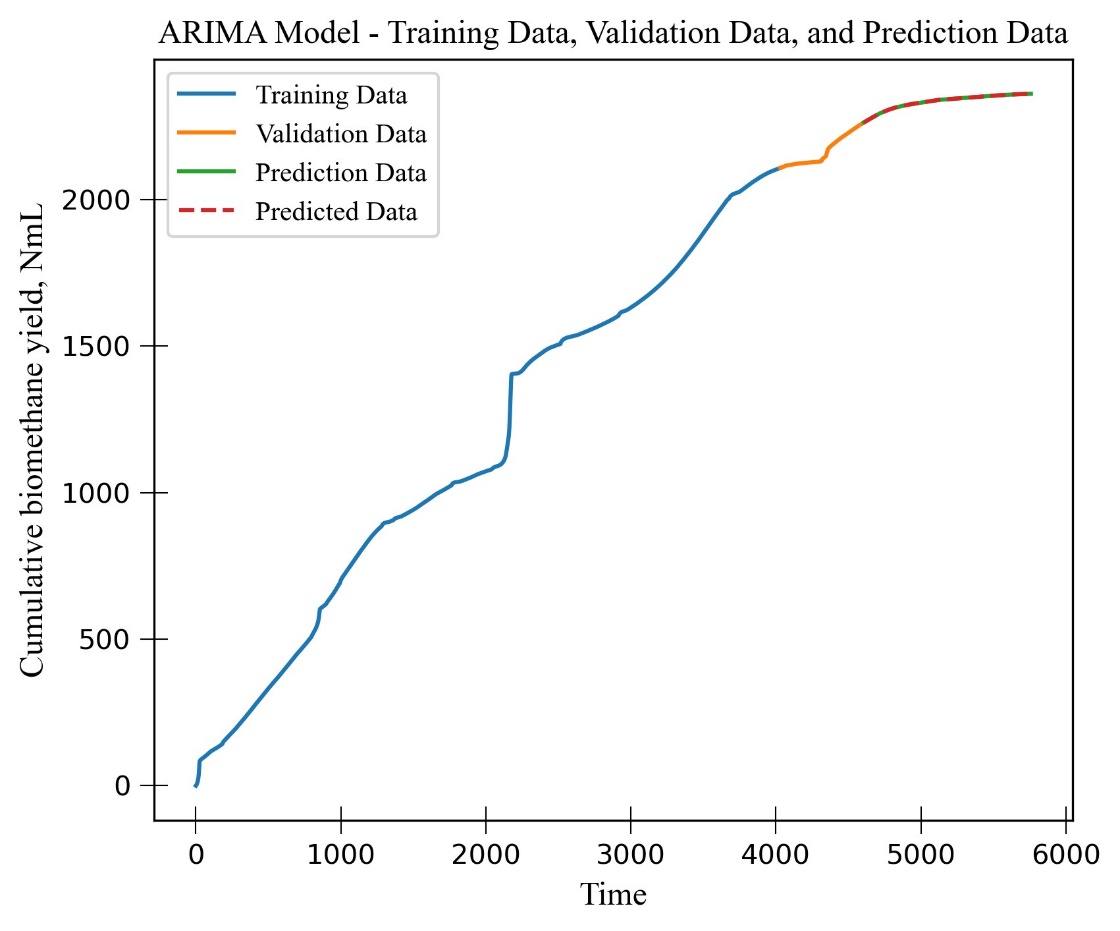


A


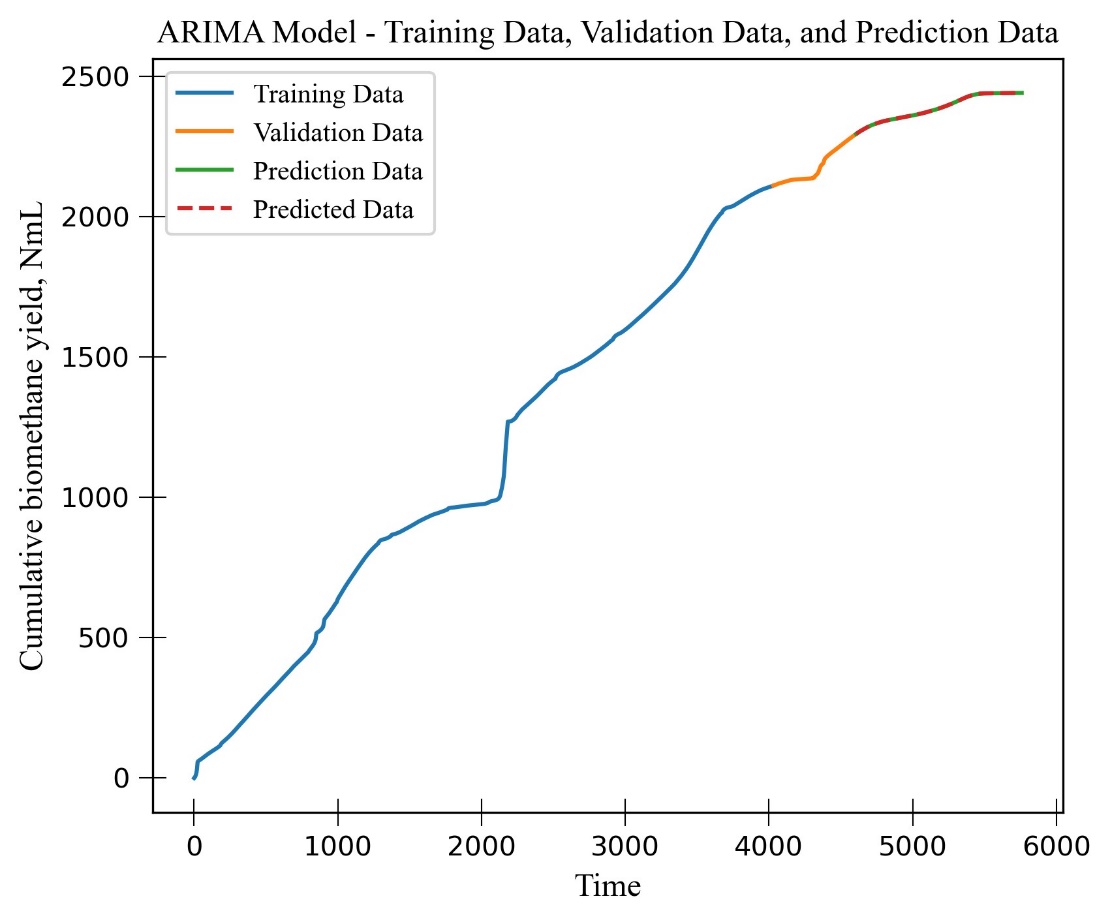


B


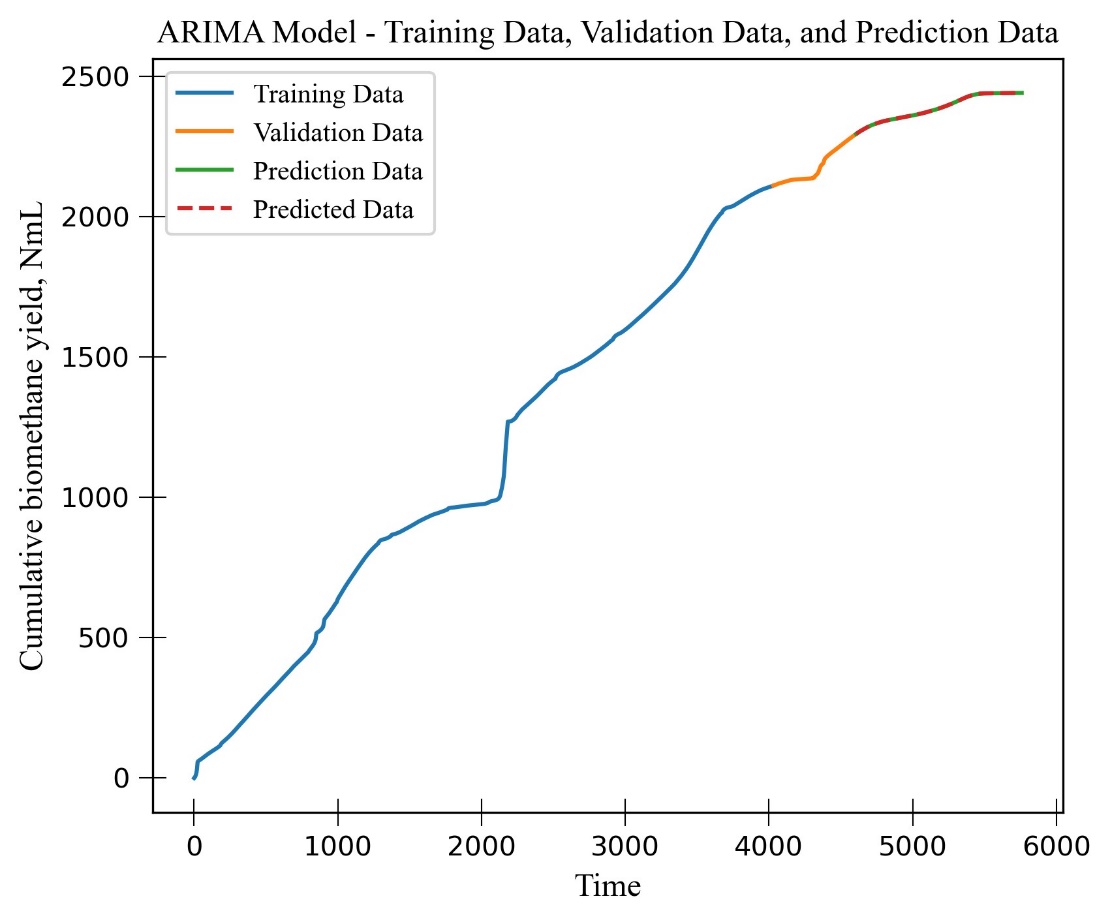


C


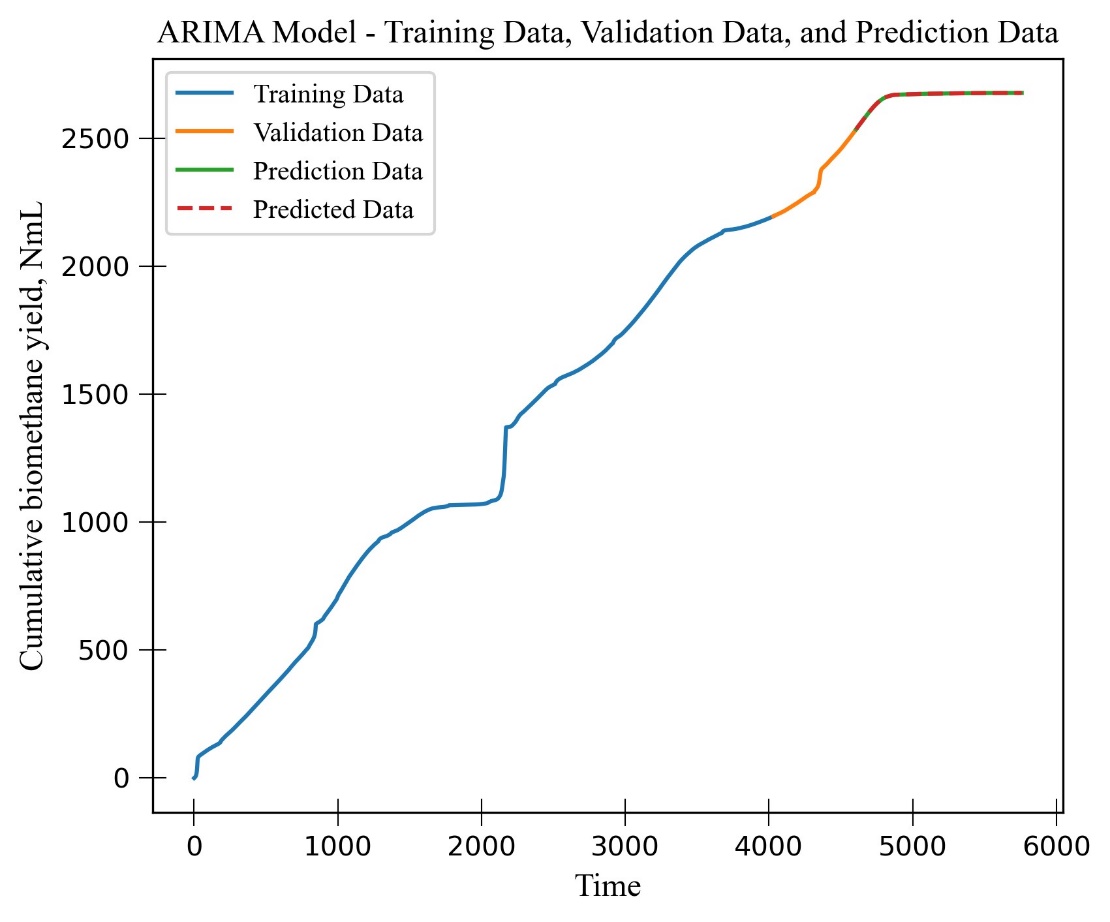


D


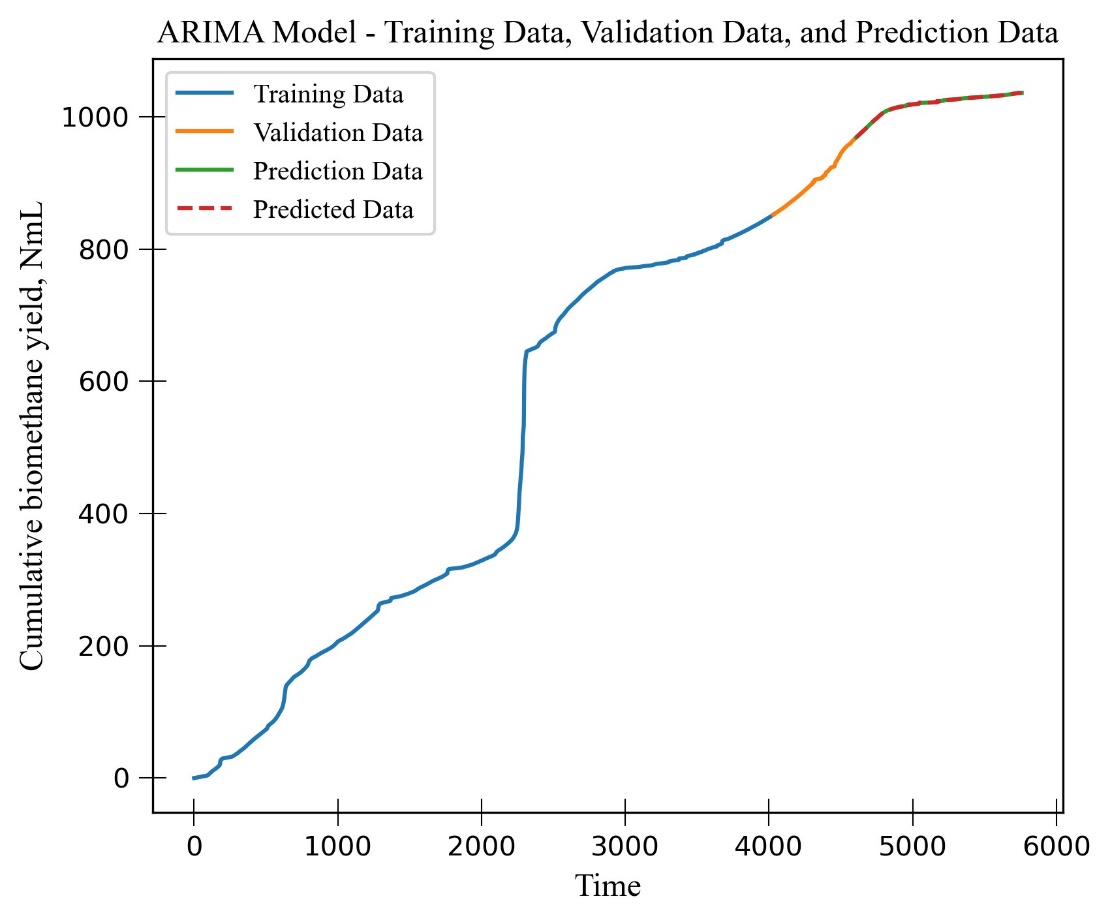


Time, [days]

0

10

20

30

40

50

60

E

Fig. S3. Cumulative biomethane yield from different dosages of biochar application in the anaerobic digestion of glucose and the corresponding fit from the autoregressive integrated moving average (ARIMA) model. A) 2.0 g/L; B) 4.0 g/L; C) 6.0 g/L; D) 8.0 g/L; and E) 0.0 g/L.

Fig. S4. The biomethane production rate from glucose influenced by the different concentrations of biochar during the first phase of the experiment.

Fig. S5. The biomethane production rate from glucose influenced by the different concentrations of biochar during the second phase of the experiment.

Fig. S6. The biomethane production rate from glucose influenced by the different concentrations of biochar during the third phase of the experiment.

Fig. S7. The biomethane production rate from glucose influenced by the different concentrations of biochar during the fourth phase of the experiment.
